# Recent progress of iron-based nanomaterials in gene delivery and tumor gene therapy

**DOI:** 10.1186/s12951-024-02550-0

**Published:** 2024-06-02

**Authors:** Ya Gong, Xiaoyan Hu, Ming Chen, Jun Wang

**Affiliations:** 1 Department of Pharmacy, The Second Affiliated Hospital of Army Medical University, Chongqing, 400037 China; 2 Institute of Biomedicine and Biotechnology, Shenzhen Institute of Advanced Technology, Chinese Academy of Sciences, Shenzhen, Guangdong 518055 China; 3 University of Chinese Academy of Sciences, Beijing, 100864 China; 4 Department of Clinical Laboratory Medicine, Southwest Hospital, Army Medical University, Chongqing, 400038 China

**Keywords:** Iron-based nanomaterials, Gene delivery, Magnetic resonance imaging, Gene therapy

## Abstract

Gene therapy aims to modify or manipulate gene expression and change the biological characteristics of living cells to achieve the purpose of treating diseases. The safe, efficient, and stable expression of exogenous genes in cells is crucial for the success of gene therapy, which is closely related to the vectors used in gene therapy. Currently, gene therapy vectors are mainly divided into two categories: viral vectors and non-viral vectors. Viral vectors are widely used due to the advantages of persistent and stable expression, high transfection efficiency, but they also have certain issues such as infectivity, high immunological rejection, randomness of insertion mutation, carcinogenicity, and limited vector capacity. Non-viral vectors have the advantages of non-infectivity, controllable chemical structure, and unlimited vector capacity, but the transfection efficiency is low. With the rapid development of nanotechnology, the unique physicochemical properties of nanomaterials have attracted increasing attention in the field of drug and gene delivery. Among many nanomaterials, iron-based nanomaterials have attracted much attention due to their superior physicochemical properties, such as Fenton reaction, magnetic resonance imaging, magnetothermal therapy, photothermal therapy, gene delivery, magnetically-assisted drug delivery, cell and tissue targeting, and so on. In this paper, the research progress of iron-based nanomaterials in gene delivery and tumor gene therapy is reviewed, and the future application direction of iron-based nanomaterials is further prospected.

## Introduction

Cancer is a kind of serious disease that threatens the health and life of human beings [1]. The strategies for tumor therapy mainly include surgical resection, chemotherapy, and radiation therapy in clinical practice. Although these methods effectively inhibit tumor growth, improve the quality of life and extend the life of patients, they also have certain limitations. For example, surgical resection is a highly effective treatment for resectable tumors in the early and middle stages. However, when once the tumor occurs metastasis and exists in various important organs and tissues, surgical resection is difficult to remove a large number of diseased tumor cells due to the consideration of avoiding the protection of various tissues and organs. Most of the chemotherapy drugs are cytotoxic substances, lack cell and tissue specificity, and may damage normal tissues while killing tumor cells [2]. In addition, long-term use of chemotherapy drugs will cause cancer cells to produce multi-drug resistance (MDR), which greatly reduces the therapeutic effect [3]. Radiation therapy can achieve targeted treatment by controlling the radiation area, thereby reducing the damage to the surrounding normal tissues. However, there are certain limitations on the dosage of radiation therapy, which makes it difficult to increase the dosage for tumors with poor radiation sensitivity. Meanwhile, radiation therapy is usually ineffective for small lesions and subclinical metastatic lesions [4]. Therefore, it is of great significance to improve the level of cancer treatment and develop new and efficient cancer treatment methods to improve human health.

**Fig. 1:**
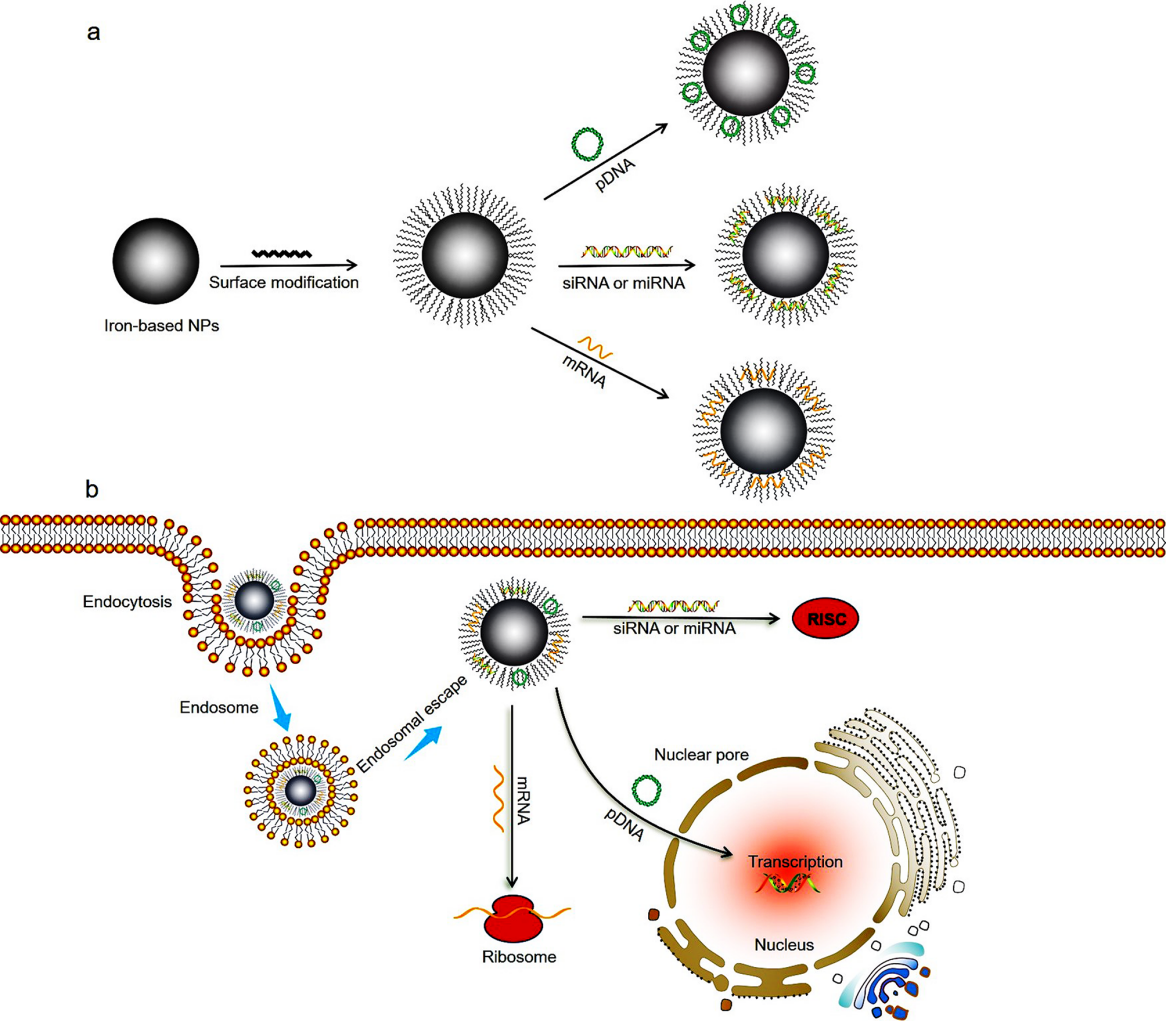
Illustration of the iron-based nanoparticles for gene delivery and tumor therapy. After surface modification, positively charged iron-based nanoparticles bind to negative charged DNA to obtain iron-based nanoparticles/genes nanocomplex via electrostatic interactions (**a**). A certain dose of the nanocomplex was injected intravenously into mice. Then, the nanocomplex can accurately target tumor tissues based on enhanced permeability and retention effect or active targeting. In tumor cell, the nanocomplex will gradually separate, and the loaded genes (such as pDNA, siRNA, mRNA and miRNA) exert anti-tumor effects (**b**) [12]

In essence, tumor is a genetic disease, and its occurrence, development, and recurrence are related to gene mutation, deletion, and deformity [5]. With the understanding and recognition for tumors at the gene molecular level, gene therapy has become the most promising treatment method among new types of cancer treatments. Gene therapy can start from the root cause of diseases, intervene in the occurrence, development, and progression of diseases by introducing therapeutic genes into target cells, correct the disorder of human gene structure or function, enhance the ability of the body to clear the diseased cells, so as to achieve the purpose of treating the disease [6–8]. Compared with other treatment methods, gene therapy can directly act on genetic material, fundamentally inhibit or block the development of diseases, and has the advantages of small side effects, good targeting, and wide applicability, which is currently a new method for tumor therapy [9, 10]. The prerequisite and essential condition for the success of gene therapy in tumor therapy is that exogenous therapeutic genes must first enter the target cells. In addition, since gene expression is strictly controlled by cells to maintain the homeostasis of normal human tissues and tumor microenvironment, the potential side effects of exogenous therapeutic genes cannot be ignored [11]. Therefore, the key to the success of gene therapy lies in selecting safe, efficient gene delivery vectors that can accurately regulate gene release and expression (Fig. 1).

**Scheme 1:**
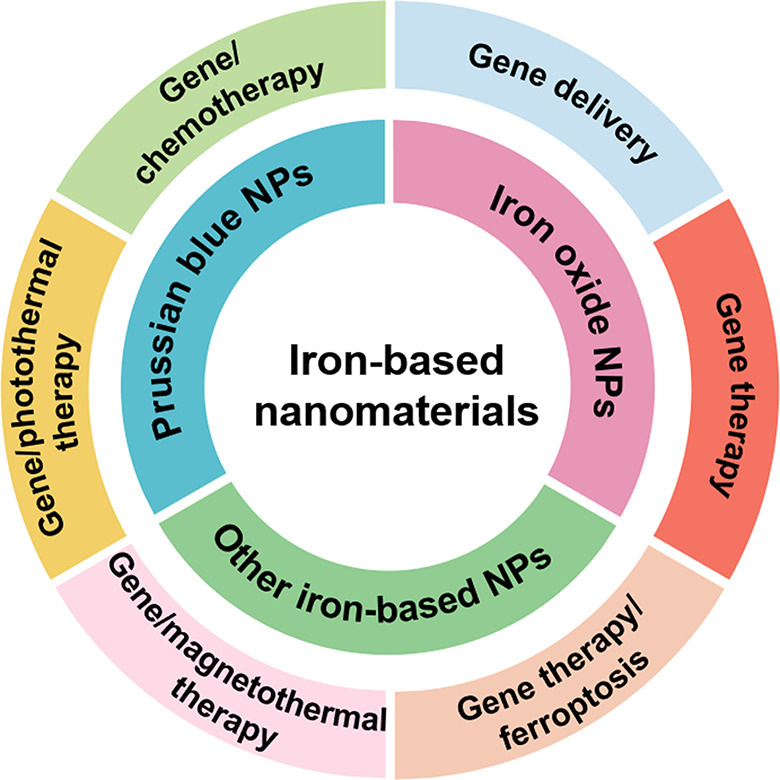
Overview of the application of iron-based nanomaterials in gene delivery and tumor gene therapy

At present, the gene delivery vectors are mainly divided into viral vectors and non-viral vectors. The transfection efficiency of viral vectors is high, but it has some disadvantages such as high immunogenicity, high toxicity, and the limited capacity for carrying genes, which leads to certain limitations in the application of viral vectors in the biological field. In contrast, non-viral vectors have attracted widespread attention due to high safety, non-immunogenicity, non-infectious, unlimited vector capacity, large-scale preparation, low cost, and targeting, which are also an indispensable class of efficient carriers in the field of gene therapy [13–16]. With the rapid development of nanotechnology, various organic and inorganic nanomaterials are used as carriers for drug and gene delivery, such as chitosan nanoparticles (NPs) [17–19], lipid-based NPs [20, 21], polymer NPs [22–24], gold NPs [25, 26], magnetic NPs [27], quantum dots (QDs) [28, 29], silica NPs [30, 31], and carbon nanomaterials [32, 33] (Table 1). Among these non-viral vectors, iron-based nanomaterials possess both nanometer effects and superparamagnetism. As a gene delivery vector, its advantages mainly include the following aspects: (1) Non-immunogenicity; (2) Iron is widely distributed in the human body, and iron-based nanomaterials also have good biocompatibility; (3) Genes can be transported to specific tissues, organs, or cells to improve the efficiency of gene delivery under the action of a magnetic field; (4) Magnetic targeting; (5) Iron-based nanomaterials have the function of magnetic resonance imaging-guided-photothermal therapy or magnetothermal therapy, achieving the integration of tumor diagnosis and therapy [34, 35]. In this review, we summarized the research progress of different types of iron-based nanomaterials in gene delivery and tumor gene therapy, and prospected the future application direction and the changes in gene therapy (Scheme 1).

**Table 1:** The advantages and disadvantages of different types of nanoparticles for gene delivery

	Nanoparticles	Advantages	Disadvantages
Organic NPs	Chitosan	Low immunogenicity; good biocompatibility; biodegradability	Low water solubility
	Lipid-based NPs	Composition-controllable; high loading capacity; low immunogenicity; hypotoxicity	Poor stability; phospholipids are easily oxidized and susceptible to the effects of metals, radiation, high temperature, pH, and enzymes; complex preparation process
	Polymer NPs	Simple production process; structure controllable; functional diversity	Easy to bind with negatively charged non-specific cells or proteins; variable cytotoxicity; low gene transfection efficiency
Inorganic NPs	Gold NPs	High specific surface area; easy surface modification and multifunctionality; easy preparation and good stability; versatility in shape design	Unstable surface structure; easy aggregation
	QDs	Good optical stability and biocompatibility	High toxicity
	Silica NPs	Adjustable pore size; easy synthesis and modification; high gene transfection efficiency; good biocompatibility	Metabolism
	Carbon nanomaterials	Low immunotoxicity; high gene transfection efficiency; high load capacity	Potential biotoxicity
	Iron-based NPs	Superparamagnetism; magnetic resonance imaging; magnetic transfection; magnetothermal therapy;	Complex preparation process; the vector alone does not have the ability of gene delivery, and surface modification is required

## The application of iron-based nanomaterials in gene delivery and tumor gene therapy

In the past few decades, iron-based nanomaterials have been widely studied due to high terrestrial abundance, unique optical and electromagnetic properties, and good biocompatibility. Iron-based nanomaterials can be divided into two main categories: (1) one is iron-based nanocrystals, including iron oxide NPs and iron-based alloys; (2) the other is iron-based nanocomposites, including amorphous iron NPs and metal-organic frames (MOFs). Based on the excellent biological functions of iron-based nanomaterials, they have been developed and widely used in the diagnosis and treatment of diseases. In this part, according to the different types of iron-based nanomaterials, the research progress is presented in gene delivery and tumor gene therapy.

### Prussian blue NPs

Prussian blue NPs (PB NPs) are inorganic NPs assembled by transition metal ions or lanthanide elements through cyanide-bridging ligand. Due to the porous structure and easy modification, PB NPs can carry drug molecules and accurately release drugs under the stimulation of the external environment, thereby improving the therapeutic efficiency of drugs. Prussian blue has specific ion exchange, adsorption, and mechanical capture characteristics. In 2003, it was approved by the US Food and Drug Administration (FDA) as an antidote for treating radioactive pollution containing thallium or cesium. In the field of medical imaging, PB NPs have strong absorption in the near-infrared (NIR) region, high photothermal conversion efficiency, and paramagnetic properties, which can be used as imaging contrast agents and tumor photothermal therapy (PTT). PB NPs also possess the properties of nanoenzymes, which can catalyze and clear excess reactive oxygen species (ROS) in the body, and are used for tumor photodynamic therapy and the treatment of ROS-related diseases [36–40]. Therefore, PB NPs have been widely used in various fields such as drug delivery, photoacoustic imaging (PAI), magnetic resonance imaging (MRI), ultrasound imaging (USI), and PTT for tumors.

#### Gene delivery

The charge transfer between Fe^3+^ and Fe^2+^ in the PB structure results in strong near-infrared (NIR) absorption in the NIR region. To take advantage of this feature, Li et al. [41] synthesized an ultrasmall chitosan-functionalized PB NPs as a non-viral gene delivery vector. The chitosan-modified PB NPs can significantly improve their stability in physiological environments and endow the NPs with a positive charge, enabling them to efficiently adsorb DNA and act as a carrier for gene transfection. In addition, the NPs have strong absorption in the near-infrared region and can convert the energy of near-infrared (NIR) light into heat, thereby enhancing gene delivery capacity, and achieving the purpose of effectively improving the efficiency of gene transfection by NIR light. In order to achieve the function of ultrasound imaging and gene transfection of tumor tissue, Li et al. [42] prepared chitosan-modified PB microbubbles (MBs@CS/PB/DNA) with stability and biocompatibility, which promoted nucleic acid delivery through the synergistic effect of ultrasound/photothermal. MBs@CS/PB/DNA microbubbles can be precisely located through ultrasound imaging. Subsequently, the microbubbles were broken by the ultrasound-targeted microbubble destruction method, and the complex of chitosan-modified PB NPs and DNA was released to reach the desired site. Under NIR irradiation, CS /PB/DNA NPs can absorb energy and improve the temperature of the cell membrane of tumor cells, thus enhancing the fluidity of the cell membrane, and further promoting more CS /PB/DNA NPs and DNA into tumor cells, improving the transfection efficiency, which was expected to be a theranostics platform for ultrasound imaging-guided tumor photothermal/gene therapy.

#### Gene/photothermal therapy

**Fig. 2:**
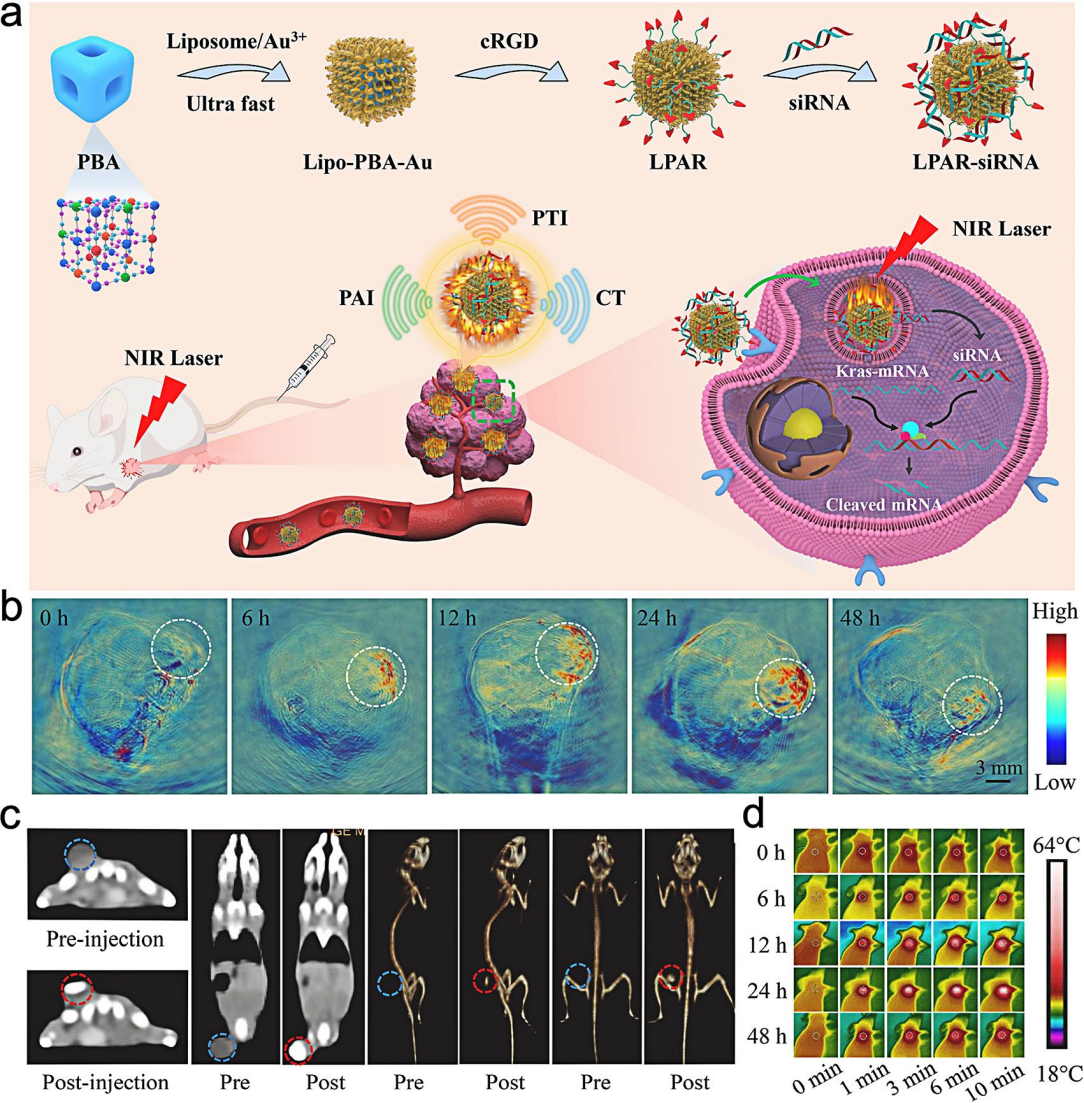
Schematic representation of Lipo-PBA-Au NPs for tumor PAI/CT imaging-guided gene/photothermal therapy (**a**); in vivo PA imaging (**b**) and CT imaging (**c**) at different time points; the infrared thermal images of the tumor under 808 nm laser irradiation (**d**). (Adapted with permission from [45]. Copyright © 2022, ELSEVIER)

Besides being gene delivery vector, PB NPs can also be used for image-guided tumor therapy. Xue et al. [43] developed a core-shell structure magnetic PB NPs modified with chitosan and pDNA, which exhibited an enhanced photothermal effect and gene transfection efficiency for tumor cells under magnetic guidance. Chen et al. [44] used PB NPs as a nanocarrier to deliver a DNA drug into cancer cells, which is a decoy oligodeoxyuncleotide (dODN) that can inhibit signal transducer and acyivator of transcripyion 3 (STAT3). The results indicated that dODN molecules had successfully entered tumor cells and could be evenly distributed in the cytoplasmic and nuclear regions in the cells. With the increase of the binding amount of dODN to PB NPs and the internalization dose of DNA-PB NPs, the killing ability of tumor cells was enhanced. Jia et al. [45] synthesized liposomes coated PB@gold nanoflower (Lipo-PBA-Au) in response to thermal stimulation using an ultrafast one-step method. Then, the RGD peptide with targeting ability was modified on the surface of liposomes to prepare the positively charged lipo-PBA-Au-RGD (LPBGD) nanogene vector. The LPBGD loaded with siRNA can effectively enter pancreatic cancer cells with high expression of αvβ3 integrin receptors. Under the irradiation of 808 nm laser, the multifunctional nanoplatform can not only achieve selective delivery and controlled release of siRNA, but also effectively convert the absorbed NIR light into heat, realizing PA/computed tomography (CT) imaging-guided gene/photothermal synergistic therapy in vitro and in vivo (Fig. 2).

**Fig. 3:**
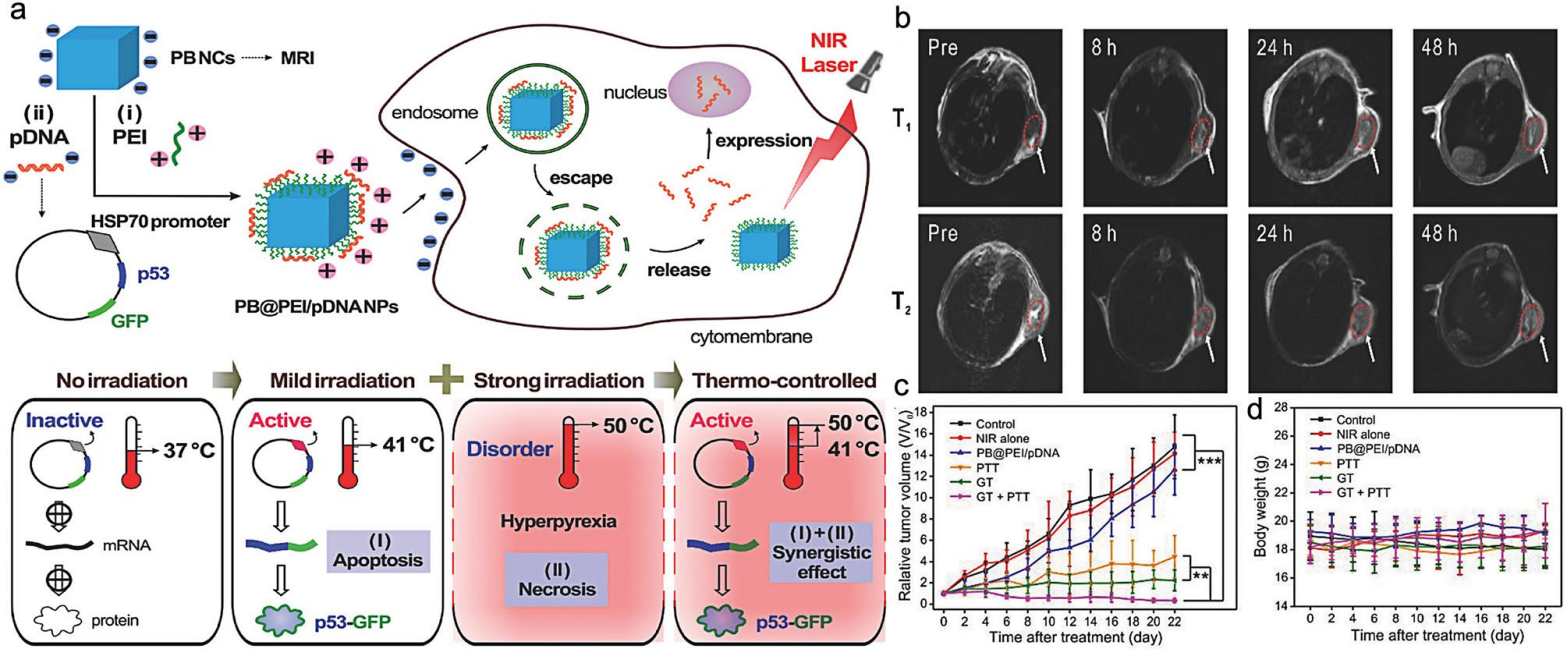
Scheme illustration of the PB@PEI/HSP70-p53-GFP NPs for gene/photothermal synergistic therapy (**a**); PB@PEI/pDNA NPs for tumor magnetic resonance imaging (**b**); the changes of tumor volume (**c**) and body weight (**d**) after different treatments. (Adapted with permission from [46]. Copyright © 2018, Wiley-VCH GmbH)

High-intensity laser (the local temperature of the tumor can reach 50 ℃) can not only kill the tumor but also damage normal organs and tissues near the tumor. To solve this problem, Chang et al. [46] designed a PB nanodiagnostic system based on the HSP70 promoter for temperature-controlled gene/photothermal synergistic therapy. In this work, HSP70 was used as the promoter and GFP was used as the reporter gene to encode the tumor suppressor gene p53. A therapeutic plasmid pDNA (HSP70-p53 GFP) was constructed and effectively loaded onto the surface of the PB NPs modified with polyethyleneimine (PEI) by electrostatic interaction. (Fig. 3). The results indicated that PB NCs loaded with plasmids showed good morphological characteristics. Under weak near-infrared laser irradiation, the generated thermal effect can induce the HSP70 promoter to regulate the expression efficiency of the target gene p53 in eukaryotic expression plasmids, thereby killing tumor cells and achieving a synergistic effect between gene therapy and photothermal therapy. In addition, the nanosystem exhibited unique T_1_/T_2_ weighted magnetic resonance imaging (MRI) performance, which was expected to achieve temperature-controlled precise treatment under MRI and provide the scientific basis for precise diagnosis and controllable treatment of tumors.

PB NPs are a highly promising nanomaterial in the biomedical field, which are widely used in gene delivery, detoxification of cesium and thallium, medical imaging, photothermal therapy, and catalytic therapy of nanoenzymes. In recent years, researchers have developed many multifunctional theranostics platforms based on PB NPs, such as PTT combined with PDT, PDT combined with drug delivery, etc. These platforms simultaneously utilize the various advantages of PB NPs and have shown good results in the diagnosis and treatment of diseases. However, in order to truly achieve the clinical application of PB NPs in tumor diagnosis and treatment, it is necessary to systematically conduct biological safety evaluations in vivo, including blood compatibility (hemolysis and coagulation), biological toxicity, distribution and metabolic in vivo, so as to provide scientific guidance for its further clinical application.

### Iron oxide NPs

#### Gene delivery

Iron oxide NPs can provide significant synergistic effects on gene therapy. Surface characteristics of iron oxide NPs not only affect the biocompatibility of NPs, but also influence gene delivery efficiency. Therefore, many organic or inorganic materials are used as coatings for iron oxide NPs to improve gene delivery efficiency. Cationic polymers are the most extensively studied non-viral gene delivery vector, which has high delivery efficiency. Polyethyleneimine (PEI) is a cationic polymer and a highly effective non-viral gene delivery vector. It is easy to bind to nucleic acids with negative charge through electrostatic adsorption, and PEI can release nucleic acids from endosomes into the cytoplasm through the proton sponge effect, ultimately targeting the nucleus. For example, Wang et al. [47] synthesized PEI coated Fe_3_O_4_ NPs to deliver siRNA to silence repressor element 1-silencing transcription factor (REST), which can efficiently suppress the proliferation and the metastasis of glioblastoma cells. Peng et al. [48] synthesized redox-responsive PEI-coated Fe_3_O_4_ NPs with good biocompatibility, high gene transfection efficiency, and enhanced MRI for controllable gene delivery. As a biodegradable material, chitosan has weaker toxicity than PEI. Stephen et al. [49] used chitosan, polyethylene glycol copolymer, and pyrocatechol to modify Fe_3_O_4_ to construct a Fe_3_O_4_-PEI-PEG gene vector system for enhancing the transfection of plasmid DNA red fluorescent protein. The results indicated that Fe_3_O_4_-PEI-PEG loaded with DNA can enhance Fe_3_O_4_ MRI and showed a significant transfection effect in vitro.

The major drawback of cationic polymers as gene delivery vectors is their high toxicity, which exhibits a dose-dependent effect. To reduce cellular toxicity, various organic and inorganic materials with good biocompatibility have been used as coating agents for Fe_3_O_4_ NPs, such as surfactants, polymers, carbohydrates, proteins, silicon, and gold. Wang et al. [50] prepared a MRI visible siRNA delivery polymer vector by amino exchange reaction between the polymer and superparamagnetic iron oxide (SPIO) NPs. In vitro experimental results indicated that siRNA can be efficiently delivered to A549 cells and effectively reduce the expression of the luciferase gene in A549 cells. After intravenous injection, EGRF-NP-SPIO can effectively accumulate at the tumor site and exert the effect of gene silencing, which was expected to be used as a substitute for bPEI25k for long-term siRNA targeted delivery and tumor therapy in vivo. Wang et al. [51] utilized polymer micelles to simultaneously load siRNA and SPIO to achieve efficient co-delivery of siRNA and SPIO within tumors. In vitro and in vivo studies shown that polymer micelles can efficiently deliver SPIO and siRNA into tumor tissues. This system expanded the delivery function of the carrier and realized the combined delivery of gene drugs and contrast agents. Thomas et al. [52] used poly(L-lysine) (PLL) to coat hyaluronic acid (HA) modified superparamagnetic iron oxide NPs for MRI-guided plasmid DNA delivery. Li et al. [53] developed core-shell magnetic silica NPs to transport VEGF shRNA. In vitro gene silencing experiments indicated that Fe_3_O_4_@SiO_2_/PEI/VEGF shRNA can downregulate the expression of the VEGF gene in MCF-7 cells.

Nano-ultrasound contrast agents have unique characteristics such as liquid gas phase transition and sonoporation, which can improve cell membrane permeability, enhance endocytosis, and promote drug uptake or gene transfection [54, 55]. Du et al. [56] used magnetic mesoporous silica NPs (M-MSNs) loaded microbubbles for plasmid DNA (pDNA) delivery by ultrasound-targeted microbubble destruction (UTMD). The NPs showed high gene-loading ability and magnetic targeting ability. The loaded microbubbles not only protect gene-loaded M-MSNs from being cleared by the reticuloendothelial system, but also enhance the pDNA transfection efficiency. Dong et al. [57] developed a core-shell structure nanodroplets composed of superparamagnetic iron oxide NPs dispersed perfluoropentane and lipid for plasmid delivery. The intracellular delivery of plasma was significantly enhanced at the tumor site through the endothelial gaps under the action of focused ultrasound and external magnetic field.

Magnetic transfection refers to inducing cell uptake and further movement toward the nucleus through an external magnetic field, which has the advantages of strong targeting and high transfection efficiency [58, 59]. Ferreras et al. [60] conducted an efficient and fast gene transfection based on magnetic NPs using magnetic fields and glycosaminoglycan (GAG)-binding enhanced transduction (GET) technology. Song et al. [61] developed a core-shell NPs composed of Fe_3_O_4_ NPs and silk fibroin for targeted delivery of c-myc antisense oligodeoxynucleotides (ODNs) into MDA-MB-231 breast cancer cells. Under magnetofection, the NPs can obtain targeted ODN delivery, achieving 70% ODN uptake efficiency within 20 min in MDA-MB-231 breast cancer cells.

#### Iron oxide NPs mediated-gene therapy

##### Gene therapy

Iron oxide NPs can not only be used as carriers for gene delivery, but also for tumor gene therapy. Mahajan et al. [62] synthesized dextran-coated superparamagnetic Fe_3_O_4_ NPs, which coupled with siPLK1 to silence the PLK1 gene and induce cell cycle arrest. In vivo MRI studies showed that the superparamagnetic NPs loaded with siPLK1 can accurately reach the tumor site and exhibit PLK1 silencing effect. The visibility of MRI enabled the spatiotemporal tracking of this therapeutic agent and guided the evaluation of the therapeutic effect of gene delivery. Zhang et al. [63] synthesized CSP/TPE@siRNA-SP94 NPs composed of cationized amylose (CA), superparamagnetic iron oxide (SPIO) NPs, and tetraphenylethylene (TPE) for liver cancer-targeted gene therapy by self-assembly method. The study showed that the prepared NPs exerted high RNA transfection efficiency and improved the uptake of Huh-7 by receptor-mediated endocytosis and electrostatic interaction. CSP/TPE@siRNA-SP94 NPs effectively accumulated at the tumor site through fluorescence imaging and MRI and significantly inhibit tumor growth. In order to solve the problem of iron oxide NPs metabolism in vivo, Peng et al. [64] constructed a Fe_3_O_4_/PEI-PEG composite nanogel with good biosafety for gene drug TGF-β1 siRNA delivery. The results of flow cytometry and confocal imaging experiments showed that the material can deliver siRNA near the nucleus. When cells were incubated with TGF-β1/complex, the expression of TGF-β1 was significantly decreased, and the growth and metastasis of S180 sarcoma cells was inhibited. In a S180 sarcoma mouse tumor model, Fe_3_O_4_/PEI-PEG composite successfully silenced the expression of TGF-β and inhibited the growth and metastasis of S180 sarcoma. Immunohistochemical analysis of tumor tissue showed that the nanocomposite reduced TGF-β1 expression, which can enhance the therapeutic effect on tumors by effectively promoting tumor tissue apoptosis. What is more, the nanogel can be gradually metabolized from the body through the kidney and lung, so as to reduce damage to normal tissues.

**Fig. 4:**
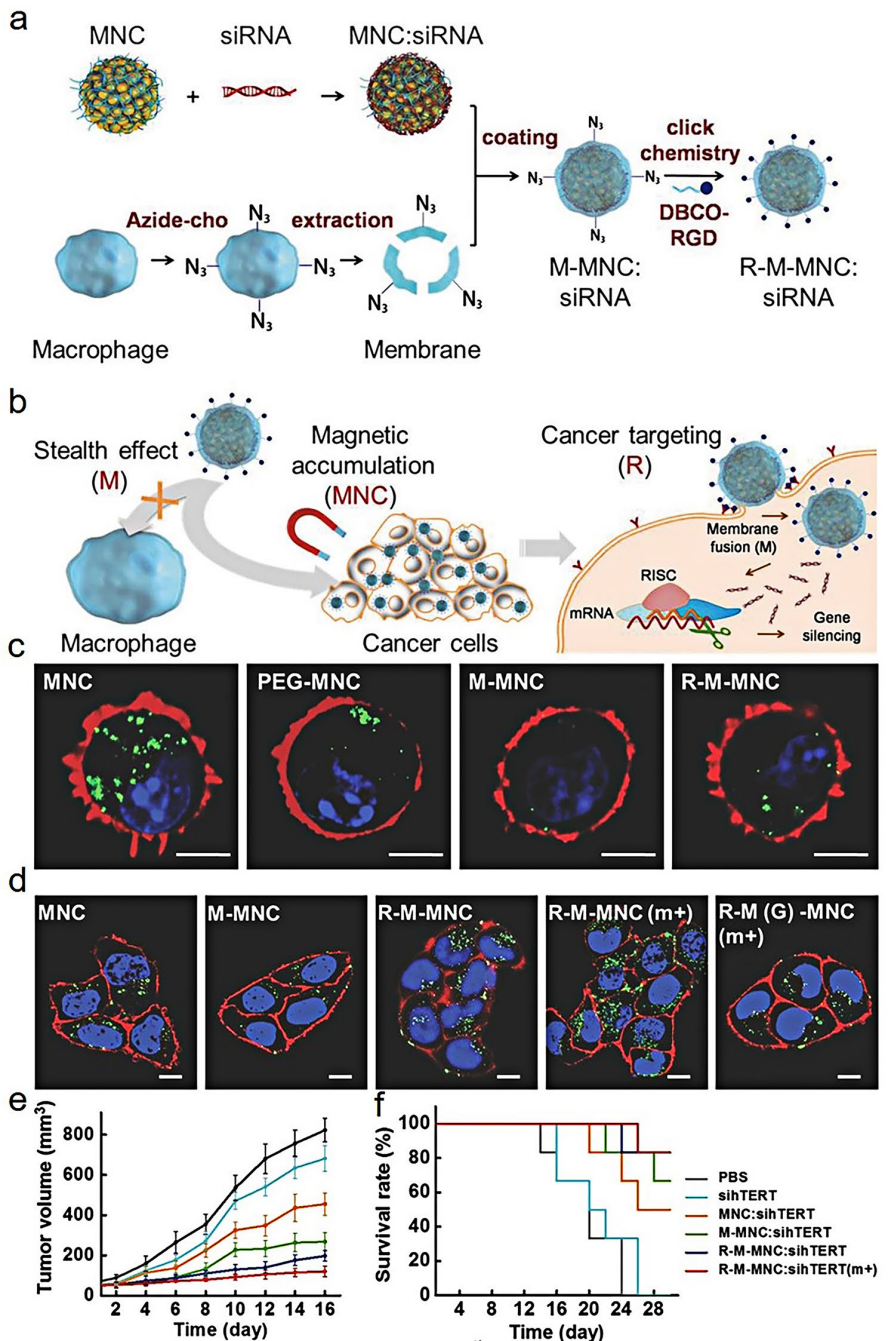
Schematic representation of the synthesis of R-M-MNC: siRNA (**a**) and its application in tumor gene therapy (**b**); the evaluation of stealth ability of different MNC-based NPs against J774A.1 macrophages (**c**); the uptake of different MNC-based NPs against MCF-7 cells (**d**); the changes of tumor volume (**e**) and survival rate of mice (**f**) after different treatments. (Adapted with permission from [69]. Copyright © 2018, Wiley-VCH GmbH)

With the emergence of new engineering strategies and a deeper understanding of natural cell functions, a novel biomimetic drug delivery system has received widespread attention and research in recent years. The multiple biological effects of the proteins on the cell membrane endows the NPs with better biocompatibility, longer blood circulation time, and immune escape performance, which has strong application potential in tumor therapy [65]. The biomimetic cell membrane drug delivery system has been successfully applied in a series of biological and biomedical research due to its special properties and excellent functions. The cell membrane extracted from the cell will retain the inherent properties of the original cell. For example, Macrophages are receiving increasing attention due to their excellent natural characteristics (non-immunogenicity, tumor cell targeting, and pathogen adhesivity) [66]. Studies have shown that macrophage membrane coated NPs can inhibit macrophage uptake and prolong the circulation time in vivo [67]. Meanwhile, macrophage membrane coated NPs also inherit the recognition characteristics of their tumor endothelial cells, which can increase the accumulation of the NPs at the tumor site [68]. Based on this, Zhang et al. [69] developed macrophage membrane-coated magnetic nanoclusters (MNCs) for siRNA-targeted delivery and high-performance anticancer therapy (Fig. 4a and b). In the cellular uptake experiment in vitro, low uptake of R-M-MNC and M-MNC was observed in J774A.1 macrophages in comparison with MNC and PEG-MNC, indicating that macrophage membrane camouflage can exert the stealth effect, which was beneficial for prolonging the circulation time of NPs in the body and avoiding being engulfed by the by the endothelial reticular system (Fig. 4c). Due to the modification of Arg-Gly-Asp (RGD), the synthesized biomimetic magnetosome enhanced cellular uptake and high gene transfection efficiency. Meanwhile, the uptake of R-M-MNC was further improved under a magnetic field (Fig. 4d). In the MCF-7-xenografted mouse model, the nanocomposite can silence the expression of target genes and significantly inhibit the growth of tumors under the action of the magnetic field (Fig. 4e and f).

Although membrane-coated gene delivery system has many advantages, there are still some problems to be solved: (1) The extraction and purification process of cells and cell membranes is more complex and requires higher technical requirements in comparison with artificially synthesized materials. (2) How to ensure the stability of cell membrane structure and the maintenance of cellular physiological functions during the preparation of nanomedicines, while avoiding bacterial infection and other contamination is the primary consideration in large-scale production of biomimetic nanomedicines. (3) Although the membrane-coated gene delivery system has good biocompatibility, its release mechanism is not yet clear at the targeted site. (4) Whether the membrane-coated NPs will cause unnecessary damage to the body or induce the expression of oncogenes, that is, the biosafety of biomimetic NPs is the most important issue they face in clinical applications. It is believed that with the deepening of the research and exploration of these issues in the scientific community, the biomimetic delivery system based on biological cell membrane will promote the precision medicine of cancer to a new stage.

##### Gene/chemotherapy

**Fig. 5:**
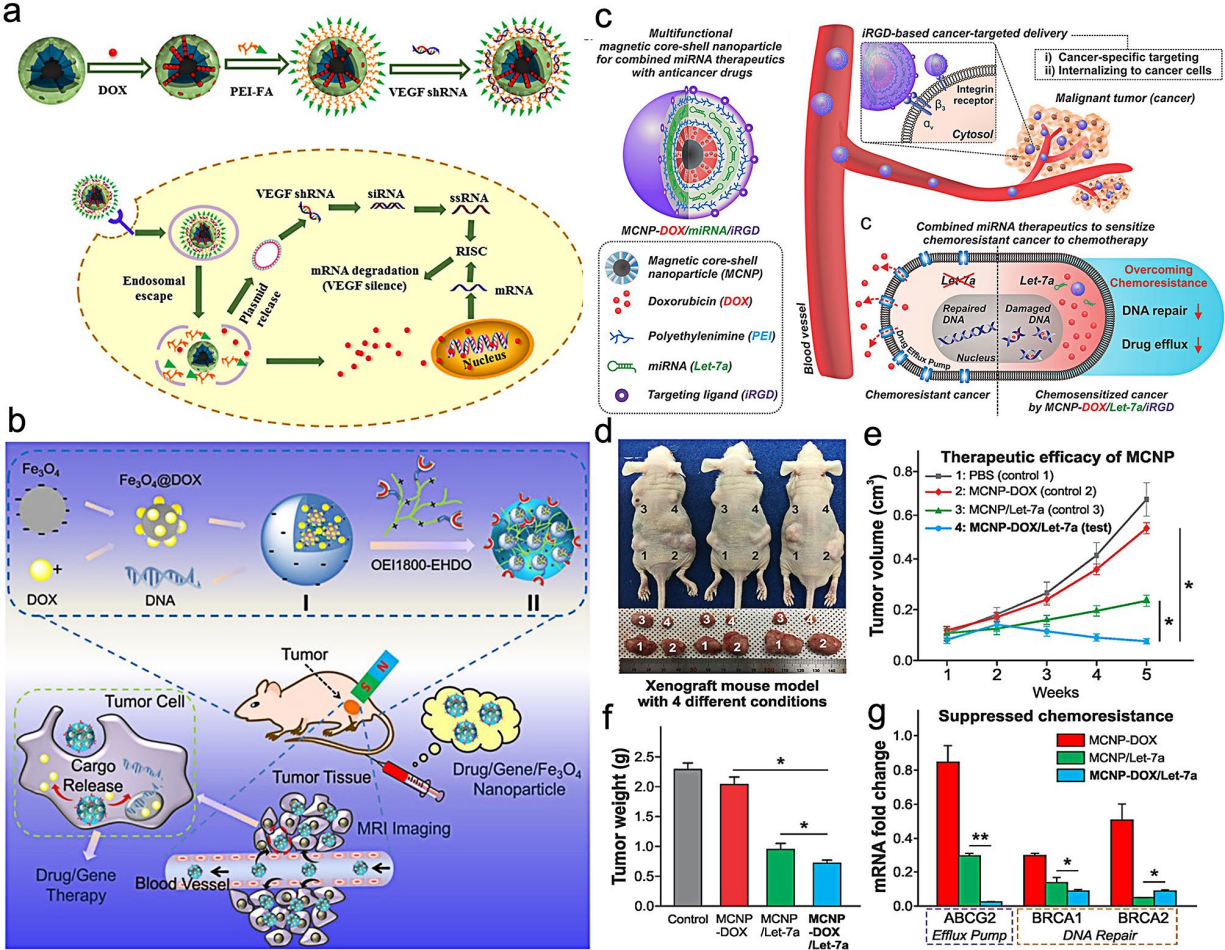
Schematic diagram of the synthesis M-MSN(DOX)/PEI-FA/VEGF shRNA and the mechanism of anti-tumor effect (**a**). (Adapted with permission from [71]. Copyright © 2016, American Chemical Society); Schematic illustration of Fe3O4@DOX/DNA/OEI1800-EHDO NPs for MRI–guided drug/gene co-delivery (**b**). (Adapted with permission from [72]. Copyright © 2016, American Chemical Society); the mechanism of MCNP-DOX/miRNA against tumor chemoresistance (**c**), the image of mice (**d**), the changes of tumor volume (**e**), tumor weight (**f**) and mRNA (**g**) under different treatments. (Adapted with permission from [74]. Copyright © 2018, American Chemical Society)

In clinical practice, chemotherapy is widely used in tumor therapy due to its simple and convenient treatment process. However, the non-specific distribution of chemotherapy drugs, toxic and side effects on normal tissues, multidrug resistance, and heterogeneity of tumors limit the potential application of chemotherapy [70]. As a result, the combination of gene and drug delivery systems has received increasing attention recently. The combination of gene therapy and chemotherapy can exert a synergistic effect, significantly improve the efficiency of tumor treatment, and reduce systemic toxic side effects, which is a promising anti-cancer strategy. For example, Li et al. [71] used magnetic mesoporous silica NPs (M-MSNs) modified by polyethylenimine-folic acid as a drug carrier to load chemotherapy drug doxycycline (DOX) and VEGF shRNA for tumor chemotherapy and gene therapy (Fig. 5a). The composite nanosystem showed high DOX loading efficiency and can effectively protect VEGF shRNA from enzymatic degradation. M-MSN(DOX)/PEI-FA NPs entered HeLa cells through folate receptor-mediated pathways and also possessed magnetic targeting ability, which can escape from lysosomes and play the role of synergistic chemotherapy and gene therapy. M-MSN(DOX)/PEI-FA/VEGF shRNA composite can effectively inhibit the expression of VEGF gene in HeLa cells, and suppress the migration, invasion and microtubule formation of endothelial cells. Jia et al. [72] developed a multifunctional nanotherapeutics system composed of Fe_3_O_4_ NPs, DNA and doxorubicin for MRI-guided tumor gene/chemotherapy (Fig. 5b). Under magnetic field (MF), the cell viability of the hybrid Fe_3_O_4_@DOX/DNA/OEI1800-EHDO (FDDP) NPs against Hela cells was significantly decreased. After intravenous injection, in the bilateral tumor-bearing model, the accumulation of NPs and the gene transfection efficiency in the MF(+) group were significantly higher than that of the MF(-) at the tumor site under the influence of a magnetic field (MF), confirming the high delivery efficiency by MF-assisted delivery strategy. Liu et al. [73] synthesized folic acid targeted magnetic nanocomplex (FA-MNP/CDDP/TFPI-2) loaded with cisplatin and gene TFPI-2. After the HNE-1 cells were transfected with TFPI-2 plasmid complex, the expression of TFPI-2 mRNA and protein in cells significantly increased. The cell growth rate and apoptosis rate of the FA-MNP/CDDP/TFPI-2 group were higher than those of the FA-MNP/CDDP and FA-MNP/TFPI-2 groups. In vivo imaging showed that FA-MNP/CDDP/TFPI-2 targeted FR-positive HNE-1 tumor, while FR-negative CNE-2 tumor did not show obvious expression of green fluorescence protein. Tunnel experiment suggested that after 23 days of tail vein injection of FA-MNP/CDDP/TFPI-2, the HNE-1 tumor in tumor bearing mice exhibited significantly cell apoptosis in comparison with the CNE-2 tumor. Combined with chemotherapy and gene therapy, the nanocomplex improved the utilization of the drug, reduced the formation of drug resistance, reduced toxic side effects, and promoted apoptosis. This magnetic nanocomposite was expected to achieve the trinity of tumor therapy, which integrated chemotherapy, gene therapy and magnetic hyperthermia. In order to overcome chemoresistance, Yin et al. [74] synthesized a multifunctional magnetic core-shell NPs (MCNP-DOX/miRNA) for let-7a microRNA(miRNA)and DOX delivery by using magnetic zinc-doped iron oxide NPs (ZnFe_2_O_4_) as a core and mesoporous silica as a shell. Under magnetofection, MCNP-DOX/miRNA NPs exhibited high delivery efficiency, about 95% (Fig. 5c). The loaded let-7a can inhibit DNA repair and drug efflux to enhance DOX-induced apoptosis via the downregulation of chemoresistance-related genes. In vivo anti-cancer results indicated the combination of gene therapy and chemotherapy showed a significantly higher rate of tumor inhibition and had no side-effect (Fig. 5d-f). Through qPCR analysis, the co-delivery of let-7a and DOX exhibited the synergistic effect of gene therapy and chemotherapy by downregulating the ABCG2, BRCA1 and BRCA2 genes-related chemoresistance (Fig. 5g).

##### Gene /photothermal therapy

**Fig. 6:**
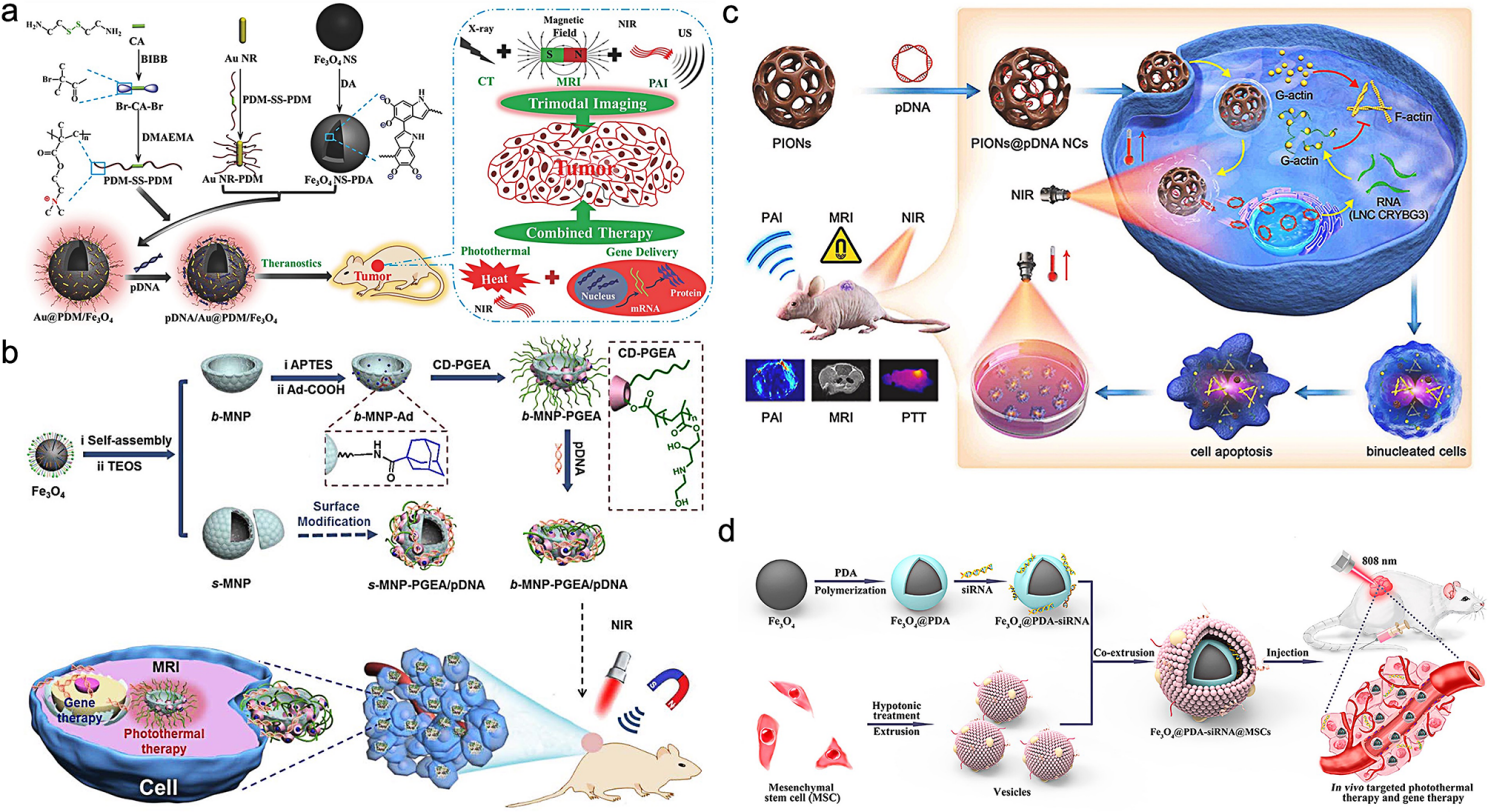
Schematic illustration of the synthesis of pDNA/Au@PDM/Fe_3_O_4_ NPs and their application in CT/MRI/PAI-guided tumor PTT/gene therapy (**a**). (Adapted with permission from [78]. Copyright © 2018, Wiley-VCH GmbH). A bowel-shaped magnetic NPs for tumor synergistic PTT/gene therapy (**b**) (Adapted with permission from [79]. Copyright © 2019, Royal Society Chemistry). Porous iron oxide NPs loaded with pcDNA3.1-LNC CRYBG3 NPs for PAI/MRI-guided tumor PTT/gene therapy (**c**). (Adapted with permission from [80]. Copyright © 2022, ELSEVIER). Mesenchymal stem cell (MSC) membrane-coated Fe_3_O_4_@PDA-siRNA NPs for tumor-targeted PTT and gene therapy (**d**). (Adapted with permission from [81]. Copyright © 2018, American Chemical Society)

Photothermal-driven gene therapy is a newly developed strategy in recent years, which is a new method to kill cancer cells and synergize gene therapy by inducing hyperthermia in tissues [75, 76]. Traditional hyperthermia is usually invasive and can cause non-specific cell damage. Nanomaterials with unique photothermal properties have been developed, which can convert light energy into heat energy under near-infrared light (NIR) irradiation, and have the advantages of stability, good biocompatibility, and easy preparation. By using such vectors to deliver therapeutic genes into cells, non-invasive photothermal therapy can be performed on tumor sites under external NIR irradiation, thus achieving a more effective anti-cancer effect. For example, Li et al. [77] synthesized a multifunctional miRNA-Fe_2_O_3_@PDA-FA drug delivery system by using Fe_2_O_3_ NPs as a contrast agent for MRI and folic acid as a targeted molecule, combined with miRNA gene therapy and polydopamine photothermal therapy. miRNA-Fe_2_O_3_@PDA-FA NPs exhibited good thermal effects in vitro and in vivo. Due to the modification of folic acid (FA), miRNA-Fe_2_O_3_@PDA-FA NPs can target osteosarcoma and enhance the accumulation of the NPs at the tumor site by MRI. The therapeutic effect of PTT combined with gene therapy was better than that of PTT alone and gene therapy alone. Hu et al. [78] developed a multifunctional polycationic AuNRs-coated Fe_3_O_4_ nanocomposites for MR/CT/PA imaging-guided tumor photothermal/gene therapy (Fig. 6a). In this system, Fe_3_O_4_ NPs were used as a contrast agent for MRI, and AuNRs can not only be used as photothermal reagents, but also as CT contrast agents. The in vitro experimental results indicated the gene transfection efficiency was higher than that of the gold-standard branched PEI. By establishing a tumor xenograft tumor model, the research results showed that the nanocomposites had good synergistic effects of PTT/gene therapy in inhibiting tumor cell proliferation, and promoting tumor cell apoptosis, which provided a new approach and strategy for the integration of tumor diagnosis and treatment.

In addition to modifying substances with photothermal properties on the surface of iron oxide NPs, the photothermal properties of iron oxide NPs themselves can also be utilized for tumor photothermal therapy. Wang et al. [79] prepared a bowl-shaped magnetic mesoporous gene delivery system by modifying cationic polymer PGEA on the surface of iron oxide nanoassemblies via host-guest interaction, which was used to construct tumor theranostic platform with gene delivery, photothermal properties, magnetic targeting, and MRI (Fig. 6b). The results showed that the gene transfection ability of the spherical bowl assembly was better than that of the spherical bowl assembly. The gene transfection ability was further enhanced when the external magnetic field was applied. When combined with PTT, the tumor growth was significantly inhibited. Huang et al. [80] used porous iron oxide nanoagents (PIONs) as a carrier for pDNA3.1 delivery that expressed long noncoding RNA (lncRNA) CRYBG3 (LNC CRYBG3) for photoacoustic and MR imaging-guided tumor photothermal/gene therapy (Fig. 6c). In combination therapy, on the one hand, the generated heat during the photothermal conversion process can be used to non-invasive ablate tumor tissue by locally heating the tissue to above 42 ℃. On the other hand, the generated heat can promote the release of LNC CRYBG3 overexpression plasmids into tumor tissue, which caused cell apoptosis by degrading the actin cytoskeleton and hindering cytoplasmic separation. The combined effects of photothermal ablation mediated by PIONs and cytotoxicity mediated by LNC CRYBG3 exhibited a synergistic tumor suppressive effect. Zhao et al. [82] constructed a multifunctional theranostic nanoplatform Fe_3_O_4_@Dex-PGEA (FDP) with MRI and gene/photothermal/magnetolytic therapy. The experimental results indicated that compared to spherical Fe_3_O_4_@Dex-PGEA (s-FDP), 1D FDP had higher gene transfection efficiency and higher endocytosis efficiency. The transfection efficiency and endocytosis could be further enhanced by applying a static magnetic field. The in vivo and in vitro experiments showed that one-dimensional Fe_3_O_4_@Dex-PGEA exhibited good MRI-guided photothermal/gene/magnetolytic synergistic effect after applying near-infrared irradiation and rotating magnetic field. It was worth noting that 1D FDP nanomaterials can be degraded and efficiently and quickly cleared by the body in acidic tumor microenvironment, which further improved the safety of the material in vivo application.

A biomimetic drug delivery system based on biofilm can simulate the functions and biological processes of human endogenous substances, accurately deliver drugs to the target location, and achieve the goal of precise treatment, which has the advantages of mild adverse reactions, good therapeutic effects, and low immunogenicity [83–85]. Inspired by this, Mu et al. [81] utilized mesenchymal stem cell (MSC) membrane-coated Fe_3_O_4_@PDA-siRNA NPs (Fe_3_O_4_@PDA-siRNA@MSC) for MRI-guided photothermal therapy and gene therapy (Fig. 6d). The prepared biomimetic NPs retained the biocompatibility of natural stem cell membrane and the ability to target tumors. The prepared nanocomplex can effectively transport siRNA into DU145 cells, inhibit the expression of endogenous Plk1 gene and promoted apoptosis. Due to the modification of MSC membrane, the Fe_3_O_4_@PDA-siRNA@MSC NPs exhibited higher accumulation at the tumor site in comparison with Fe_3_O_4_@PDA NPs by MRI. The synergistic combination of gene therapy and PTT can significantly inhibit tumor growth in vivo.

**Fig. 7:**
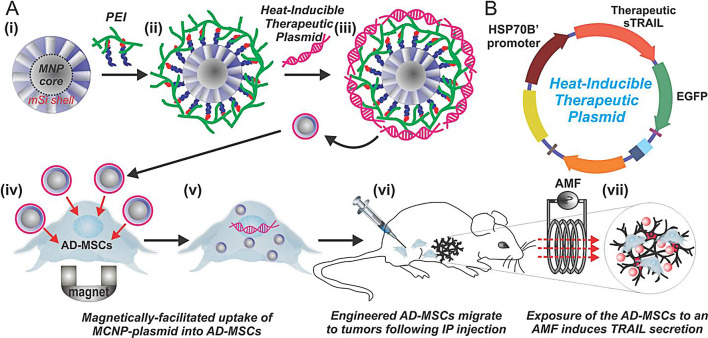
Illustration of a heat-activated stem cell-based gene therapy using magnetic hyperthermia (**a**). (Adapted with permission from [86]. Copyright © 2016, ELSEVIER)

##### Gene/magnetothermal therapy

Magnetothermal therapy (MHT) refers to the magnetic loss generated by magnetic materials in an alternating magnetic field, which leads to the generation of heat in the material and raises the temperature of the surrounding area, achieving the effect of killing tumor cells [87–89]. When iron oxide NPs are accumulated at the tumor site, MH can only occur in the tumor area without damaging normal tissue. MHT has the advantages of high selectivity, high biocompatibility, and deep tissue penetration [90, 91]. Yin et al. [86] synthesized mesoporous magnetic NPs as a gene carrier to deliver and activate heat-inducible therapeutic plasmid (Fig. 7). By introducing the MCNP-plasmid into adipose-derived mesenchymal stem cells (AD-MSCs), the expression of TNF-related apoptosis-inducing ligand (TRAIL) was regulated under alternating magnetic hyperthermia. In vivo studies indicated that the growth of tumor was significantly inhibited in comparison with the control group. Li et al. [92] synthesized shMUC1-C@ Fe_3_O_4_ magnetic NPs for triple-negative breast cancer gene/magnetic hyperthermia therapy. The shMUC1-C@ Fe_3_O_4_ NPs can efficiently silence the MUC1-C gene and exert a good magneto-thermal effect. The generated loco-regional moderate hyperthermia directly killed tumor cells by targeting MUC1-C under the action of alternating magnetic field. Meanwhile, the Fe^2+^ in the shMUC1-C@Fe_3_O_4_ NPs induced apoptosis and ferroptosis by upregulating the expression level of BCL-2-associated X protein (Bax) and cleaved-caspase3 and inhibiting the expression level of GPX4, NRF2, and FTH1. In vivo results indicated the tumor growth was significantly inhibited under the synergistic effect of gene/magnetic hyperthermia therapy. Wang et al. [93] developed a liver cancer theranostic system based on magnetic-mesoporous silica NPs, which achieved the synergistic release of suicide genes and prodrugs in cells and magnetic resonance imaging, magnetic targeting, and magnetic hyperthermia-enhanced suicide gene therapy for liver cancer. M-MSNs exhibited uniform morphology, excellent superparamagnetism, high drug loading capacity, and high gene transfection efficiency. M-MSNs exhibited a high-efficiency magnetothermal conversion effect under the action of alternating magnetic fields. Suicide gene therapy based on M-MSNs showed a certain anti-liver cancer effect at the cellular level and animal level. When combined with magnetic targeting and magnetothermal therapy, the efficacy of gene therapy had been significantly improved.

##### Ferroptosis-gene therapy

Ferroptosis, as a new non-apoptotic pathway of cell death, has shown broad prospects in the treatment of cancer. The characteristic of ferroptosis is the accumulation of reactive oxygen species (ROS) and lipid peroxidation products, which are sufficient to cause cell death [94–96]. As is well known, iron plays an important role in the production of ROS in Fenton reactions, which can induce ferroptosis. In addition, the downregulation of glutathione peroxidase 4 (GPX4) or inhibition of cysteine/glutamate reverse transporters can prevent clearance of lipid peroxidation reactions and exacerbate the process of ferroptosis [97]. Various iron-based nanomaterials have been widely used in ferroptosis, and iron-based nanomaterials can accumulate at tumor sites through passive or active targeted delivery. Due to the acidity of the tumor microenvironment, iron released from iron-based nanomaterials can participate in inducing ferroptosis [98]. Based on this, researchers have combined ferroptosis with gene therapy for tumor treatment. Hu et al. [99] developed a combination of superparamagnetic iron oxide nanoworms and polyethylenimine-NH_2_ (PEN) as a carrier for small interfering Snail2 RNA (siSnail2) delivery. Combined with low-dose cisplatin, the multifunctional NPs can induce ferroptosis in liver tumor cells, inhibit the epithelial-tomesenchymal transition (EMT) phenomenon of tumor cells and suppress or weaken the metastasis and invasion of liver cancer cells. Gao et al. [100] developed a gene-interfered ferroptosis therapy (GIFT) for tumor by combining gene interference tools (CRISPR/Cas13a and miRNA) with iron NPs. The results indicated that GIFT can induce apoptosis of various cancer cells, including 3 types of blood tumor cells and 15 types of solid tumor cells. However, the same treatment had no significant effect on the seven normal cells. Moreover, the levels of reactive oxygen species (ROS) and iron content in cells treated with GIFT were significantly increased, indicating that the killing effect of GIFT was mediated by the elevated ROS levels caused by the increase of intracellular iron content. The results of anti-tumor experiments in vivo showed that polyethylenimine (PEI) coated Fe_3_O_4_ NPs can not only serve as delivery carriers for gene interference tools to knockdown the levels of FPN and LCN2, but also as donors of iron ions to participate in the Fenton reaction, accumulate ROS to induce tumor cell death, and inhibit tumor growth, which provided new directions for developing more effective tumor gene therapy.

Iron oxide NPs have unique physicochemical properties and can be used as contrast agents for magnetic resonance imaging, which successfully combine tumor diagnosis and gene therapy, and promote the birth of gene diagnosis and therapy. In addition, iron oxide NPs can greatly improve the therapeutic effect of tumors by utilizing magnetic transfection and hyperthermia. As various technologies become more mature, iron oxide NPs will open a new chapter in tumor gene diagnosis and therapy. However, there are still many problems that need to be further studied, such as the tumor targeting of most iron oxide NPs needs to be improved. Due to the complex environment in vivo, ions, serum proteins, and blood cells all affect the effectiveness of iron oxide NPs. Therefore, further exploration is needed to determine the optimal physicochemical properties of iron oxide NPs, such as surface charge, type of coating, and immune responses.

### Other types of iron-based NPs

#### Iron-based metal organic frameworks

Metal organic frameworks (MOFs) are a newly emerging class of highly ordered crystalline porous materials composed of inorganic metal centers (metal ions or metal clusters) and organic ligands [101, 102]. MOFs containing iron elements can not only release ferrous/iron ions at the tumor site to induce ferroptosis, but their pore size structure can also effectively load different types of drugs, which has unique advantages in the field of iron death synergistic therapy.

Recent studies have shown that the p53 gene can not only regulate cell cycle, apoptosis, and aging, but also inhibit cysteine uptake and induce cell ferroptosis by inhibiting the expression of SLC7A11 [103, 104]. Zheng et al. [105] encapsulated the p53 plasmid into a metal organic framework (MON-p53) and utilized the dual effect of ferroptosis and apoptosis induced by the p53 gene to treat the tumor. Researchers combined tannic acid (an FDA-approved food additive extracted from green tea) with Fe^3+^ to form metal organic frameworks (MOFs) on the surface of the PEI/p53 plasmid complex (PEI/p53), thereby obtaining MON-p53. Once MON-p53 was internalized by cells, Fe^3+^ can induce a Fenton reaction to produce ROS, and high concentration ROS led to the accumulation of PUFA-LPs-OOH. Meanwhile, the expressed p53 protein further inhibited the elimination of lipid peroxides. After MON-p53 treatment, the morphological changes of human fibrosarcoma HT1080 cells indicated tumor cells undergo ferroptosis. After intravenous administration, MON-p53 significantly inhibited the growth of HT1080 tumors, and the median survival time was significantly extended from 44.5 days (PBS-treated mice) to 75 days (MON-p53-treated mice).

#### Iron-based metal coordinated nanomaterials

**Fig. 8:**
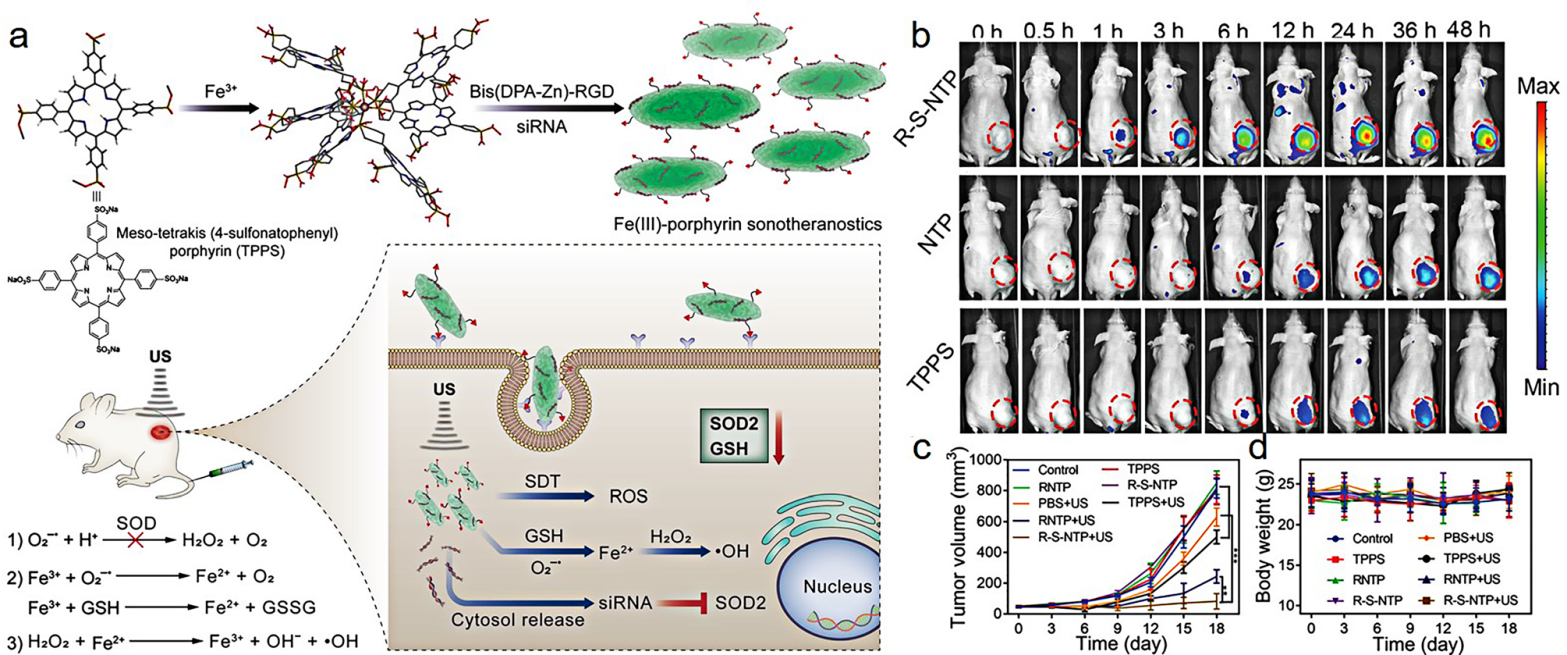
Schematic diagram of the synthesis and anti-tumor mechanism of Fe(III)-porphyrin sonotheranostics (**a**); in vivo fluorescence imaging of tumor at different time points after intravenous administration of TPPS, NTP, and R-S-NTP (**b**); the changes of tumor volume (**c**) and body weight (**d**) after different treatments. (Adapted with permission from [107]. Copyright © 2019, Wiley-VCH GmbH)

Due to the unique properties of iron ions, self-assembled NPs can be obtained by chelating with polymers or polyphenol derivatives. Ma et al. [106] designed a novel PAMAM dendrimer coordinated Fe (III) complex delivery the plasmid (p53 pDNA) containing human tumor suppressor gene p53 and green fluorescent protein EGFP gene for ferroptosis/gene therapy of PANC-1 tumor. On the one hand, the synthesized Fe-Au-DENP-HQ can effectively release Fe (III) in the tumor acidic microenvironment and undergo a Fenton-like reaction with H_2_O_2_ in the microenvironment, which resulted in the generation of reactive oxygen species, the accumulation of lipid peroxides (LPO), and the depletion of glutathione (GSH), thus inducing iron-dependent ferroptosis of tumor cells. On the other hand, the p53 pDNA in the complex upregulated the activity of PTEN and p53 while downregulating the activity of SLC7A11 and GPX-4 in tumor cells. This not only directly triggered apoptosis but also further enhanced ferroptosis in cancer cells through positive regulation. In the pancreatic cancer mouse model, the Fe-Au-DENP-HQC/p53 complex exerted a combined therapeutic effect of ferroptosis/gene therapy, which significantly inhibited tumor growth without causing significant damage to other organs in the mouse. Zhu et al. [107] designed and synthesized nano-sonosensitizers that can enhance the therapeutic effect of sonodynamic therapy. Nano-sonosensitizers were prepared by using metal iron ions and sound sensitizer molecules. The modification of polypeptides targeting liver cancer tissue can enhance the drug accumulation at the tumor site. The loaded siRNA specifically downregulated the intracellular antioxidant enzyme SOD2, which further enhanced the effect of sonodynamic therapy. The Fe^3+^ in the NPs not only downregulated the intracellular antioxidant GSH, but also further underwent a cascade Fenton reaction to produce hydroxyl radicals, both of which can enhance the effectiveness of sonodynamic therapy (Fig. 8a). In vivo fluorescence imaging results indicated that the accumulation of drugs in the tumor of each group increased rapidly and reached peak at 24 h postinjection. The fluorescence signal of the tumor in the R-S-NTPR group was significantly higher than that in the TPPS and NTP group. This is because the EPR effect of NPs and the active targeting effect of RGD on liver cancer cells play a major role in the enhanced accumulation effect (Fig. 8b). The combination of sonodynamic therapy and gene therapy can significantly inhibit the growth of tumor, and be used be for drug tracing and therapeutic monitoring by fluorescence imaging and magnetic resonance imaging (Fig. 8c and d).

#### Iron-doped NPs

**Fig. 9:**
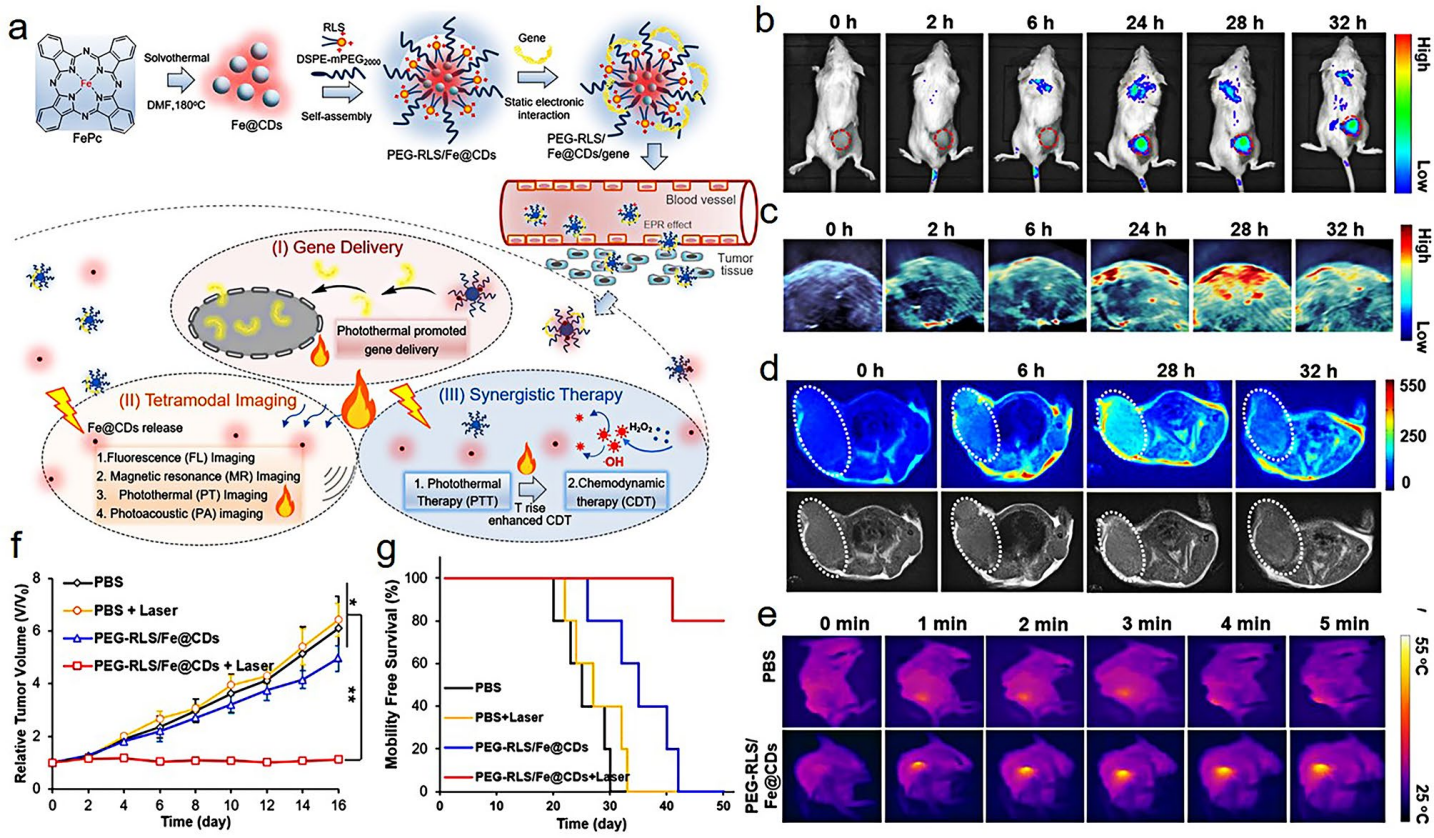
Schematic representation of the synthesis iron-doped carbon dots and its application in fluorescence/MR/PA imaging-guided tumor PTT and enhanced chemodynamic therapy (**a**); in vivo fluorescence (**b**), photoacoustic (PA) imaging (**c**) and T_1_-weighted magnetic resonance imaging (**d**) of tumor at different time points after intravenous administration of PEG-RLS/Fe@CDs; the infrared thermal images of the tumor under 660 nm laser irradiation at a density of 0.5 W/cm^2^ (**e**); the changes of tumor volume of tumor (**f**) and survival rate of mice (**g**) after different treatments. (Adapted with permission from [108]. Copyright © 2021, ELSEVIER)

Luo et al. [108] synthesized an iron-doped carbon dots nanohybrids (PEG-RLS/Fe@CDs) for fluorescence/MR/PA imaging-guided tumor gene/photothermal/chemodynamic therapy (Fig. 9a). The doping of iron significantly improved the photothermal conversion efficiency of the NPs, which resulted in that the gene delivery efficiency was increased by 3.5-fold and 2-fold in vitro and in vivo, respectively. In the 4T1 tumor-bearing mice model, the prepared PEG-RLS/Fe@CDs can conduct fluorescence/PA/MR teramodal imaging-guided tumor photothermal/chemodynamic synergistic therapy (Fig. 9b and e). In vivo studies confirmed the bioactivity of PEG-RLS/Fe@CDs as a nanoenzyme significantly inhibited tumor growth and prolonged the survival rate of mice (Fig. 8f and g).

#### FePS_3_ NPs

**Fig. 10:**
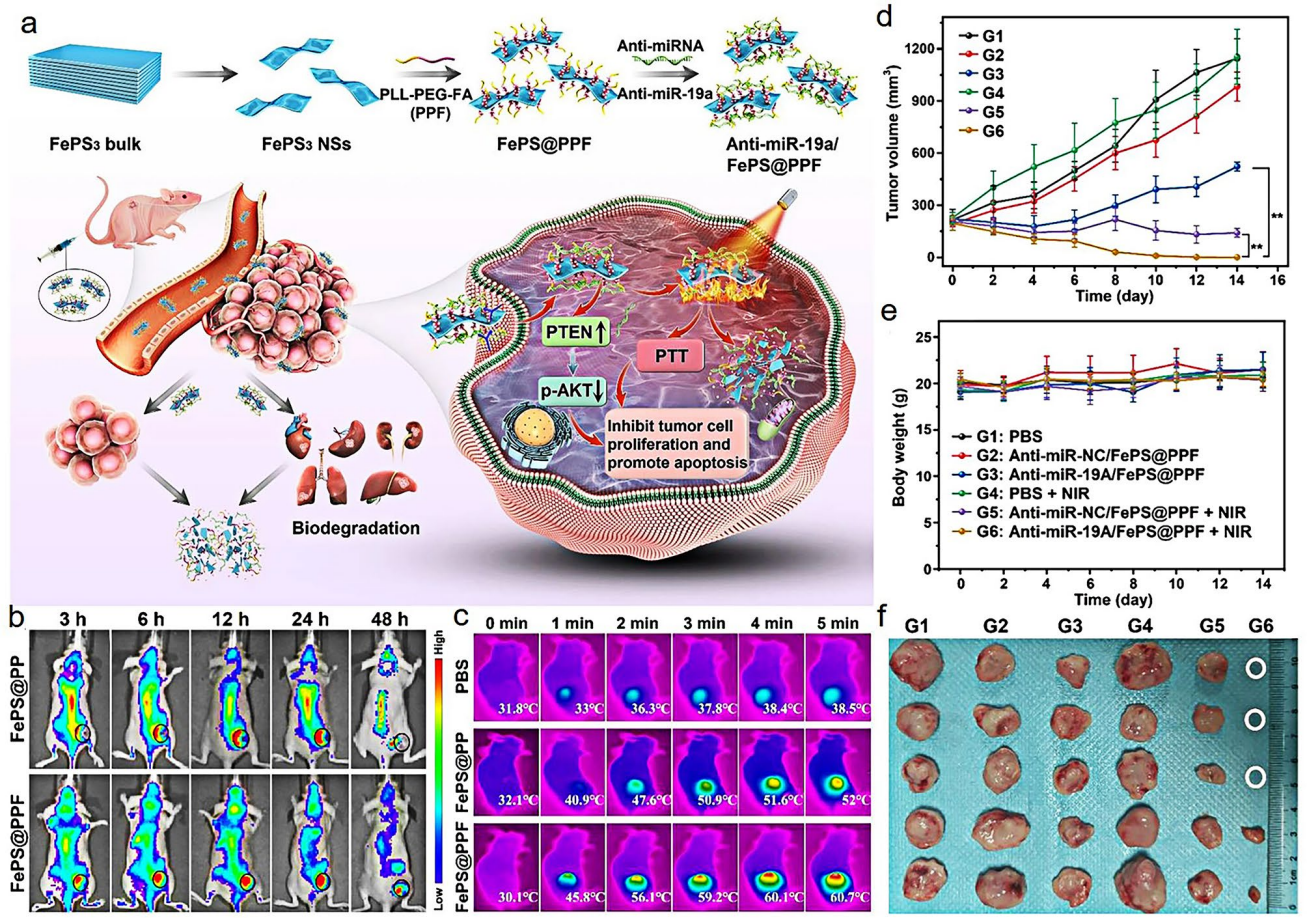
Schematic illustration of the fabrication process and anti-tumor mechanism of anti-miR-19a/FePS@PPF nanosheets (**a**); in vivo fluorescence imaging of tumor at different time points after intravenous administration of FePS@PP and FePS@PPF NPs (**b**); the infrared thermal images of the tumor under 1064 nm laser irradiation at a density of 1.0 W/cm^2^ (**c**); the changes of tumor volume (**d**), body weight (**e**) and photographs of tumors (**f**) after different treatments. (Adapted with permission from [109]. Copyright © 2023, Springer Nature)

Luo et al. [109] developed poly-L-lysine-PEG-folic acid (PPF) modified two-dimensional (2D) FePS_3_ nanosheets (PPF@FePS_3_) as a carrier to deliver miR-19a inhibitor for osteosarcoma gene and NIR-II photothermal therapy (Fig. 10a). PPF@FePS_3_ NPs can effectively deliver miR-19a inhibitor to osteosarcoma cells and induce the upregulation of PTEN protein and downregulation of p-AKT protein. After intravenous injection, the accumulation of FePS@PPF NPs was higher than that of FePS@PP NPs at the tumor site by fluorescence imaging due to the modification of folic acid (Fig. 10b). The combined therapy can effectively eliminate osteosarcoma in comparison with single therapy (Fig. 10c and f). More importantly, the PPF@FePS_3_ can be metabolized from the body, thereby reducing long-term toxicity to normal tissues.

### Patents survey and clinical trails

With unique physicochemical properties, iron-based nanomaterials can achieve obvious antitumor effects through gene delivery and gene therapy. To date, there are kinds of ongoing clinical studies of non-viral DNA based therapeutics have been applied for tumor therapy (Table 2). Meanwhile, different types of iron-based nanomaterials have achieved substantial advancements in the field of biomedical engineering, particularly in tumor imaging and therapy (Table 3). What is exciting is that some iron-based nanomaterials have already entered the clinical stage and have even been successfully used in clinical applications, approved the US Food and Drug Administration (FDA). Currently, this hot field has been drawing researcher’s attention. More and more patents based on iron-based nanoparticles for gene delivery have been applied (Table 4). However, to successfully complete the clinical translation, a number of obstacles still need to be overcome. This indicates our topic review hold certain degree of foresightedness.

**Table 2:** Some of ongoing clinical studies of non-viral DNA-based tumor therapy

NCT Number	Drugs	Indications	Sponsor	Phase
NCT03502785	INO-5401 + INO-9012	Urothelial Carcinoma	INOVIO Pharmaceuticals	Phase 1 and 2
NCT03491683	INO-5401 + INO-9012	Glioblastoma	INOVIO Pharmaceuticals	Phase 1 and 2
NCT03603808	VGX-3100	Anal Intraepithelial Neoplasia, Human Papillomavirus-16 Positive, Human Papillomavirus-18 Positive	AIDS Malignancy Consortium	Phase 2
NCT05191784	GX-I7	Recurrent Glioblastoma	Genexine, Inc.	Phase 2
NCT03444376	GX-188E	Cervical Cancer	Genexine, Inc.	Phase 1 and 2
NCT04015700	GNOS-PV01 + INO-9012	Unmethylated Glioblastoma	Washington University School of Medicine	Phase 1
NCT05771584	AST-301	Gastric Cancer	Aston Sci. Inc.	Phase 2
NCT05163223	AST-301	Breast Cancer	Aston Sci. Inc.	Phase 2
NCT05794659	AST-201	Ovarian Cancer	Aston Sci. Inc.	Phase 2

**Table 3:** Some of ongoing clinical studies of iron-based nanomaterials for tumor imaging and therapy

NCT Number	Drugs	Indications	Sponsor	Phase
NCT02033447	Magnablate I	Thermoablation-Retention and Maintenance in the Prostate	University College London Hospitals	Early Phase 1
NCT02751606	Ferumoxtran-10	Rectal and Breast cancer	Radboud University Medical Center	Phase 3
NCT00188695	Ferumoxtran-10	Prostate (cT1-cT3) or bladder cancer	University Health Network, Toronto	Phase 1 and 2
NCT04682847	Ferumoxytol injection	Primary & Metastatic Hepatic Cancers	Allegheny Singer Research Institute	Not Provided
NCT05092750	Magnetic Particle-ICG	Lymph Node Mapping in Colorectal Cancer	M.D. Anderson Cancer Center	Phase 1 and 2
NCT06271421	NanoTherm	Adjuvant Therapy of Glioblastoma Multiforme	Poznan University of Medical Sciences	Not Provided
NCT04484909	NBTXR3	Radiation Therapy for the Treatment of Locally Advanced or Borderline-Resectable Pancreatic Cancer	M.D. Anderson Cancer Center	Phase 1
NCT02442531	CriPec® docetaxel	Docetaxel Given to Patients With Solid Tumours	Cristal Therapeutics	Phase 1

**Table 4:** Patents enlisting the gene delivery with iron-based nanoparticles

Patent number	Publishing date	Nanoparticles	Integrated component	Reference
US11305024 B2	Apr 2022	Iron oxide NPs	siRNA	[110]
US20210154323 A1	May 2021	Iron oxide NPs	Peptide/protein	[111]
US20200146995 A1	May 2020	Iron oxide NPs	Protein/nucleotides	[112]
US10568970 B2	Feb 2020	USPIO	Nucleic acid	[113]
US10286073 B2	May 2019	Magnetic NPs	CRISPR/Cas9	[114]
EP2396039 B1	Aug 2018	Superparamagnetic iron oxide NPs	pDNA with IL-12 gene	[115]
US9675714 B1	Jun 2017	Superparamagnetic iron oxide NPs	DNA	[116]
US9649388 B2	May 2017	Magnetic NPs	Double-stranded oligo RNA	[117]
US9050362 B2	Jun 2015	PEI-PAAIO	pDNA	[118]
KR101313180 B1	Sep 2013	Magnetic NPs	Nucleic acid	[119]
KR20130045078	May 2013	Magnetic NPs	Gene	[120]
US2012156686 A1	Jun 2012	MNP-SiO_2_	DNA/antibodies	[121]
KR20110105568 A	Sep 2011	Iron oxide NPs	DNA/RNA	[122]
US07846201 B2	Dec 2010	Magnetic NPs	Nucleic acid/pDNA	[123]
US7459145 B2	Dec 2008	Magnetic NPs	Nucleic acid/peptide	[124]
US20070059775 A1	Mar, 2007	Iron oxide NPs	Peptide	[125]
WO2006055447 A2	July 2006	USPIO	Oligonucleotide	[126]

**Table 5:** A summary of different types of iron-based nanomaterials in gene delivery and tumor gene therapy

Type	Materials	Delivered gene	Method of therapy	Cancer cell/tumor model	Ref.
Prussian blue NPs	CS/PB NPs	pDNA	--	HeLa cells	[41]
	MBs@CS/PB NPs	pEGFP-C1 plasmid	--	HeLa cells	[42]
	Fe_3_O_4_@PB@CS NPs	pDNA	gene/PTT	HeLa cells	[43]
	MUA-PB NPs	dODN	gene/PTT	22rv1 human prostate carcinoma epithelial cells	[44]
	Lipo-PBA-Au-cRGD	siRNA	gene/PTT	Human pancreatic cancer cells	[45]
	PB@PEI NPs	HSP70	gene/PTT	HeLa cells	[46]
Iron oxide NPs	PEI-coated Fe_3_O_4_ NPs	siRNA	--	glioblastoma cells	[47]
	IONs@rPEI NPs	pDNA	--	HeLa cells	[48]
	IOCCP-PEI	DNA	--	SF767 human glioblastoma multiforme cells	[49]
	PAME-SPIO NPs	siRNA	--	A549 cells	[50]
	PEAE/SPIO NPs	siRNA	--	U87 cells	[51]
	PLL-HA-SPION NPs	pDNA	--	murine colon cancer cells	[52]
	Fe_3_O_4_@SiO_2_/PEI/VEGF	shRNA	--	MCF-7 cells	[53]
	M-MSN@MBs NPs	pDNA	--	ovarian carcinoma cells	[56]
	PMUNDs	pEGFP-C1	--	SMMC-7721 cells	[57]
	Dextran-MNP	pDNA	--	NIH3T3 cells	[60]
	MSPPs	ODNs	--	MDA-MB-231 breast cancer cells	[61]
	StAv-SPIONs	siPLK1	gene therapy	6606PDA cells	[62]
	CSP/TPE-SP94	siRNA	gene therapy	Huh-7 ​cells	[63]
	Fe_3_O_4_/PEI-PEG NGs	TGF-β1 siRNA	gene therapy	S180 cells	[64]
	R-M-MNC	siRNA	gene therapy	MCF-7 cells	[69]
	M-MSN(DOX)/PEI-FA	VEGF shRNA	gene/chemotherapy	HeLa cells	[71]
	SPIONs	pDNA	gene/chemotherapy	HeLa cells	[72]
	FA-MNP/CDDP	TFPI-2 plasmid	gene/chemotherapy	HNE-1 cells	[73]
	magnetic zinc-doped iron oxide NPs	let-7a microRNA	gene/chemotherapy	MDA-MB-231 cells	[74]
	FA-Fe_2_O_3_@PDA NPs	miR-520a-3p	gene/PTT	osteosarcoma cells	[77]
	Au@pDM/Fe_3_O_4_ NPs	pDNA	gene/PTT	C6 cells	[78]
	b-MNP-PGEA	pDNA	gene/PTT	4T1 cells	[79]
	PIONs	pcDNA3.1-LNC CRYBG3	gene/PTT	A549 cells	[80]
	Fe_3_O_4_@Dex-PGEA NPs	pDNA	gene/PTT	4T1 cells	[82]
	Fe_3_O_4_@PDA@MSCs	siRNA	gene/PTT	293T cells	[81]
	ZnFe_2_O_4_ magnetic NPs	heat-inducible TRAIL plasmid	gene/MHT	Human ovarian cancer cells	[86]
	MUC1-C@Fe_3_O_4_ NPs	shRNA	gene/MHT	TNBC cells	[92]
	R-M-MSNs-P	HSV-TK/GCV	gene/MHT	HepG2 cells	[93]
	PEN-coated SPIONs	Snail2 RNA	ferroptosis	A549 cells	[99]
	FeNP	CRISPR/Cas13a- and miRNA	gene-interfered ferroptosis therapy	WEHI-3 cells	[100]
Other types of iron-based NPs	Metal organic network	p53 plasmid	ferroptosis	4T1 cells	[105]
	Fe-Au DENP-HQC	p53-pDNA	apoptosis and ferroptosis	PANC-1 cells	[106]
	Fe(III)/TPPS	siRNA	SDT	HepG2 cells	[107]
	Fe@CDs	DNA	PPT/CDT	4T1 cells	[108]
	FePS_3_ nanosheets	anti-miR-19a	gene/PTT	HOS cells	[109]

## Conclusion and outlook

Iron is one of the essential nutrients for living organisms, which plays a very important role in immune function, oxygen transportation, energy metabolism, and DNA synthesis and repair, etc. In this review, we systematically summarize the latest research progress of iron-based nanomaterials in gene delivery and tumor gene therapy in recent years based on the different types of iron nanomaterials (Table 5). The unique physicochemical properties of iron-based nanomaterials have shown enormous potential in biomedical fields such as magnetic targeted gene delivery, tumor magnetic hyperthermia, and ferroptosis. As the vector of gene delivery, iron-based nanomaterials can intelligently mediate external magnetic fields and achieve high-efficient gene transfection under external magnetic field. Meanwhile, based on the characteristics of easy modification and functionalization of iron-based nanomaterials, on the basis of giving full play to the passive targeting of iron-based nanocarriers, the targeting of tissues and cells, and the MRI-guided tumor therapy can be achieved by improving the size, morphology and physicochemical properties of iron-based nanocarriers or modifying the specific targeting molecules. Although significant progress has been made in gene delivery and tumor gene therapy based on iron-based nanomaterials, there is still a long way to go to realize the clinical application, and a series of challenges need to be overcome: (1) Delivery efficiency. High-efficient and non-toxic gene delivery is crucial for iron-based nanomaterials in tumor gene therapy. However, it remains a challenge to ensure that nanomaterials can accurately and efficiently reach tumor sites in complex biological environments. Based on this, one approach is based on the unique physical environment of tumors, such as abnormal tumor blood vessels, high osmotic pressure, and abnormal tumor microenvironment, the delivery efficiency and the accumulation of NPs are enhanced at the tumor site through some physical methods (heat, ultrasound, magnetic fields, etc.) or chemical methods (enzymes, pH, cytokines, etc.). The second approach is to modify targeting ligands. There are a large number of specific or highly expressed receptors on the surface of tumor cells. By modifying ligands on gene non-viral vectors, the specific recognition and binding of ligands and receptors can be used to increase the uptake of NPs by tumor cells, thus enhancing the effect of gene therapy. (2) Biosafety. Studying the biosafety of nanomaterials remains a top priority in achieving their clinical use. For the non-clinical safety study of vector, in addition to focusing on their own toxicity, it is also necessary to investigate the long-term toxicity, carcinogenicity, immunogenicity, and immunotoxicity caused by the vector. The toxicity caused by nano size, the binding of the vector to plasma proteins, blood compatibility, and the structure-activity or toxicity relationships caused by the effects of various components within the NPs should be emphasized. In addition, the direct toxic reactions caused by iron accumulation and iron overload after long-term treatments, as well as indirect toxic reactions such as oxidative stress, DNA damage, and normal tissue cell apoptosis induced by iron ions, are all challenges faced by iron-based nanomaterials. How to improve diagnosis and treatment effect while reducing potential adverse reactions requires extensive preclinical study based on a detailed explanation of the delivery mechanisms in vivo and their interactions with complex biological environments. (3) Mechanism: Currently, research mainly focuses on how iron-based vectors can carry genes into cells. There are few reports on the process of genes in cells and the mechanism of how they enter the nucleus. (4) The stability of gene expression and regulation: The key to gene therapy lies in the precise regulation of specific gene expression. However, iron-based nanomaterials may be affected by the in vivo environment during gene delivery, leading to gene inactivation or unstable expression. In addition, how to ensure the sustained and stable expression of genes in tumor cells is also a problem that needs to be solved in clinical translation.

In summary, the clinical translation issues of iron-based nanomaterials in the field of gene delivery and tumor gene therapy involve multiple aspects and require interdisciplinary cooperation and efforts to solve. Through in-depth research and technological innovation, it is expected to overcome these obstacles and promote breakthroughs in the clinical application of iron nanomaterials in the field of gene delivery and tumor gene therapy.

## Data Availability

No datasets were generated or analysed during the current study.
